# MEK inhibitor–based genomically matched combinatorial targeted therapies in metastatic pancreatic adenocarcinoma with *KRAS* alterations

**DOI:** 10.1093/oncolo/oyag040

**Published:** 2026-02-17

**Authors:** Elizabeth D Auckley, Stephanie Yohay, Grace Ying, Aniko Szabo, Jacquelyn Wittmann, Justin Grahl, Mohammed Aldakkak, Samih Z Thalji, Thomas McFall, Razelle Kurzrock, Ben George, Mandana Kamgar

**Affiliations:** Department of Medicine, Medical College of Wisconsin, Milwaukee, WI 53226, United States; Department of Medicine, Medical College of Wisconsin, Milwaukee, WI 53226, United States; Department of Medicine, Division of Hematology and Oncology, Medical College of Wisconsin, Milwaukee, WI 53226, United States; Data Science Institute, Division of Biostatistics, Medical College of Wisconsin, Milwaukee, WI 53226, United States; Department of Medicine, Medical College of Wisconsin, Milwaukee, WI 53226, United States; Department of Pharmacy, Froedtert Health, Milwaukee, WI 53226, United States; Department of Surgery, Medical College of Wisconsin and the LaBahn Pancreatic Cancer Program, Milwaukee, WI 53226, United States; Department of Surgery, Medical College of Wisconsin and the LaBahn Pancreatic Cancer Program, Milwaukee, WI 53226, United States; Department of Biochemistry, Medical College of Wisconsin and the LaBahn Pancreatic Cancer Program, Milwaukee, WI 53226, United States; Department of Medicine, Division of Hematology and Oncology, Medical College of Wisconsin and the LaBahn Pancreatic Cancer Program, Milwaukee, WI 53226, United States; Department of Medicine, Division of Hematology and Oncology, Medical College of Wisconsin and the LaBahn Pancreatic Cancer Program, Milwaukee, WI 53226, United States; Department of Medicine, Division of Hematology and Oncology, Medical College of Wisconsin and the LaBahn Pancreatic Cancer Program, Milwaukee, WI 53226, United States

**Keywords:** pancreatic ductal adenocarcinoma, MEK inhibitors, targeted therapy, KRAS

## Abstract

**Introduction:**

Pancreatic ductal adenocarcinoma (PDAC) is often caused by mutations in multiple genes, including *KRAS* (activating the Ras-Raf-MEK-ERK pathway). This study evaluated the role of MEK inhibitor (MEKi)–based combinatorial targeted therapies in patients with PDAC.

**Methods:**

This is a retrospective/prospective observational, single-institution study, including 29 patients with metastatic PDAC with *KRAS* alterations, treated with MEKi therapies between 2022 and 2024.

**Results:**

Ten patients had *KRAS* G12R (34.5%), ten G12D (34.5%), and nine G12V (31%). The majority of patients received MEKi therapy as third-line and beyond (KRAS G12R/G12D/G12V: 60%/50%/78%, respectively). Median overall survival from MEKi initiation for *KRAS* G12R/G12D/G12V was 8.2/5.1/4.7 months (*P* = .5), respectively, and median progression-free survival was 4.4/2.3/1.4 months (*P* = .11). Six (21%) patients discontinued at least one drug in the treatment combination due to toxicity.

**Conclusions:**

MEKi–based combinatorial therapies had modest disease control in patients with *KRAS* G12R, and minimal disease control in patients with *KRAS* G12D/V in the late-line setting.

Implications for Practice MEKi–based genomically matched individualized combinatorial targeted therapies in patients with metastatic PDAC with *KRAS* alterations had modest benefit, which was most prominent in patients with *KRAS* G12R (5/10 (50%) disease control >4 months, 3/5 (60%) treated as first-line in the metastatic setting). If patients are treated with such alterations, it is best done in a center with an available molecular tumor board and precision oncology experts with the ability to find the best individualized therapy at any given time and to manage the toxicities associated with such combinations.

## Introduction

Up to 95% of pancreatic ductal adenocarcinoma (PDAC) cases are driven by oncogenic alterations in the *KRAS* gene, most commonly *KRAS* G12D (40%), *KRAS* G12V (30%), and *KRAS* G12R (20%) [1]. These mutations lead to activation of the Ras-Raf-MAPK kinase (MEK)–extracellular signal-related kinase (ERK) pathway, resulting in cancer initiation and propagation [2]. MEK inhibitors alone, or with chemotherapy, have provided suboptimal results in unselected populations with PDAC [3]. This is partly because *KRAS* alterations, nearly in all cases, are also accompanied by additional alterations in tumor suppressors, such as *TP53*, *CDKN2A/B*, and *SMAD4,* which create a complex network of drivers, resulting in early resistance if unopposed [3]. Potential for matching targeted therapies to these genomic alterations is present for these genes: *TP53* matched to vascular endothelial growth factor (VEGF)/VEGF receptor (VEGFR) inhibitor [4]; *CDKN2A/B* matched to cyclin dependent kinase (CDK) 4/6 inhibitor [5]; *SMAD4* matched to MEK inhibitor (MEKi) +/− epidermal growth factor receptor (EGFR) or pan-human epidermal growth factor receptor (HER) inhibitors [6, 7]. The overarching question in this study was the utility of matching targeted therapies to multiple genomic alterations at once in each individual (individualized matched therapies). This study investigated the efficacy of MEKi–based genomically matched individualized combinatorial targeted therapies in patients with metastatic PDAC with *KRAS* alterations.

## Materials and methods

This study was a retrospective review of records of patients retrospectively/prospectively enrolled in the institutional review board-approved protocols (PRO00045718[NCT05630989]; PRO00044894[NCT05802069]) at a single, tertiary care academic center with precision oncology expertise. Adults with metastatic PDAC treated with MEKi combinatorial therapies (excluding MEKi plus hydroxychloroquine) between March 2022 and September 2024 were included. The choice of treatment was made by the treating physician, based on suggestions of the Medical College of Wisconsin molecular tumor board. Next-generation sequencing was performed by Clinical Laboratory Improvement Amendments certified commercial vendors on tissue in all patients.

Targeted therapies were started at 50% of the standard drug dose in 2-drug combination therapies, and 33%-50% of the standard dose when three or more drugs were combined, to decrease toxicities. If a specific drug led to a certain toxicity in a combination therapy, that drug alone was interrupted/discontinued, and other drugs were continued. Only toxicities which were at least possibly related to any of the combinatorial targeted therapies and led to treatment interruptions or discontinuations are reported.

Overall survival (OS) and progression-free survival (PFS) from the time of MEKi initiation were estimated using the Kaplan-Meier method. The Log-rank test was used for comparison of OS and PFS between patients with different *KRAS* alterations (G12R, G12D, G12V). Analyses were performed using R 4.3.1 (R Foundation for Computing, Vienna, Austria).

## Results

Twenty nine patients were included in the study: ten (34.5%) *KRAS* G12D, ten (34.5%) G12R, and nine (31%) G12V mutations (Table 1; Supplementary Table 1). Median age (years) of patients at MEKi therapy initiation was 67, and 59% were female. Demographic characteristics were not significantly different between patients based on *KRAS* alterations (G12R/G12D/G12V). For metastatic PDAC disease, 60% of patients with G12R alteration, 50% with G12D, and 78% with G12V received MEKi therapy as third-line or beyond. Most matched alterations: treatments were: *KRAS*: MEKi (*KRAS* alterations in *N* = 29/29: matched to trametinib *N* = 25/29 [86%], cobimetinib *N* = 5/29 [14%]); *TP53*: VEGF/VEGFR inhibitor (*TP53* alterations in *N* = 24/29 [83%]: matched to bevacizumab *N* = 12/24 [50%]); *CDKN2A*/B: CDK4/6 inhibitor (*CDKN2A* alterations in *N* = 18/29 [62%]: matched to palbociclib *N* = 12/18 [67%] and abemaciclib *N* = 2/18 [10%]); *SMAD*4: MEKi+/-EGFR or pan-HER inhibitor (alterations in *N* = 9/29 [31%]: matched to MEKi in *N* = 9/9 plus afatinib [pan-HER inhibitor] in *N* = 3/9 [33%] and erlotinib [EGFR inhibitor] in *N* = 3/9 [33%]).

**Table 1:** Demographic and treatment characteristics of patients.

Characteristic	***KRAS* G12R** *N* = 10^a^	***KRAS* G12D** *N* = 10^a^	***KRAS* G12V** *N* = 9^a^	*P*-value
**Sex**				.57^b^
** F**	6 (60%)	7 (70%)	4 (44%)	
** M**	4 (40%)	3 (30%)	5 (56%)	
**Age at MEK inhibitor initiation**	68 (61–74)	66 (45–80)	67 (48–78)	.84^c^
**Lung only metastasis at first metastatic/advanced diagnosis**				.16^b^
** N**	8 (80%)	10 (100%)	6 (67%)	
** Y**	2 (20%)	0 (0%)	3 (33%)	
**Tumor location**				.11^b^
** Body**	6 (60%)	1 (10%)	2 (22%)	
** Head**	2 (20%)	6 (60%)	6 (67%)	
** Tail**	2 (20%)	3 (30%)	1 (11%)	
**Metastatic/advanced at initial diagnosis**				.89^b^
** N**	5 (50%)	6 (60%)	6 (67%)	
** Y**	5 (50%)	4 (40%)	3 (33%)	
**ECOG at MEKi–based treatment initiation**				.26^b^
** 0**	4 (40%)	2 (20%)	1 (11%)	
** ≥1**	6 (60%)	8 (80%)	8 (89%)	
**Line of treatment for MEKi for advanced disease**				.094^b^
** 1**	4 (40%)	1 (10%)	1 (11%)	
** 2**	0 (0%)	4 (40%)	1 (11%)	
** ≥3**	6 (60%)	5 (50%)	7 (78%)	
**Evaluable response by imaging on MEKi–based**				.32^b^
** N**	2 (20%)	3 (30%)	0 (0%)	
** Y**	8 (80%)	7 (70%)	9 (100%)	
**Best response clinical investigator**				.64*^b^*
** Progressive disease**	3 (30%)	5 (50%)	5 (56%)	
** Stable disease**	7 (70%)	5 (50%)	4 (44%)	

a *n* (%); Median (Min–Max).

b Fisher’s exact test.

c Kruskal-Wallis rank sum test.

**Figure 1:**
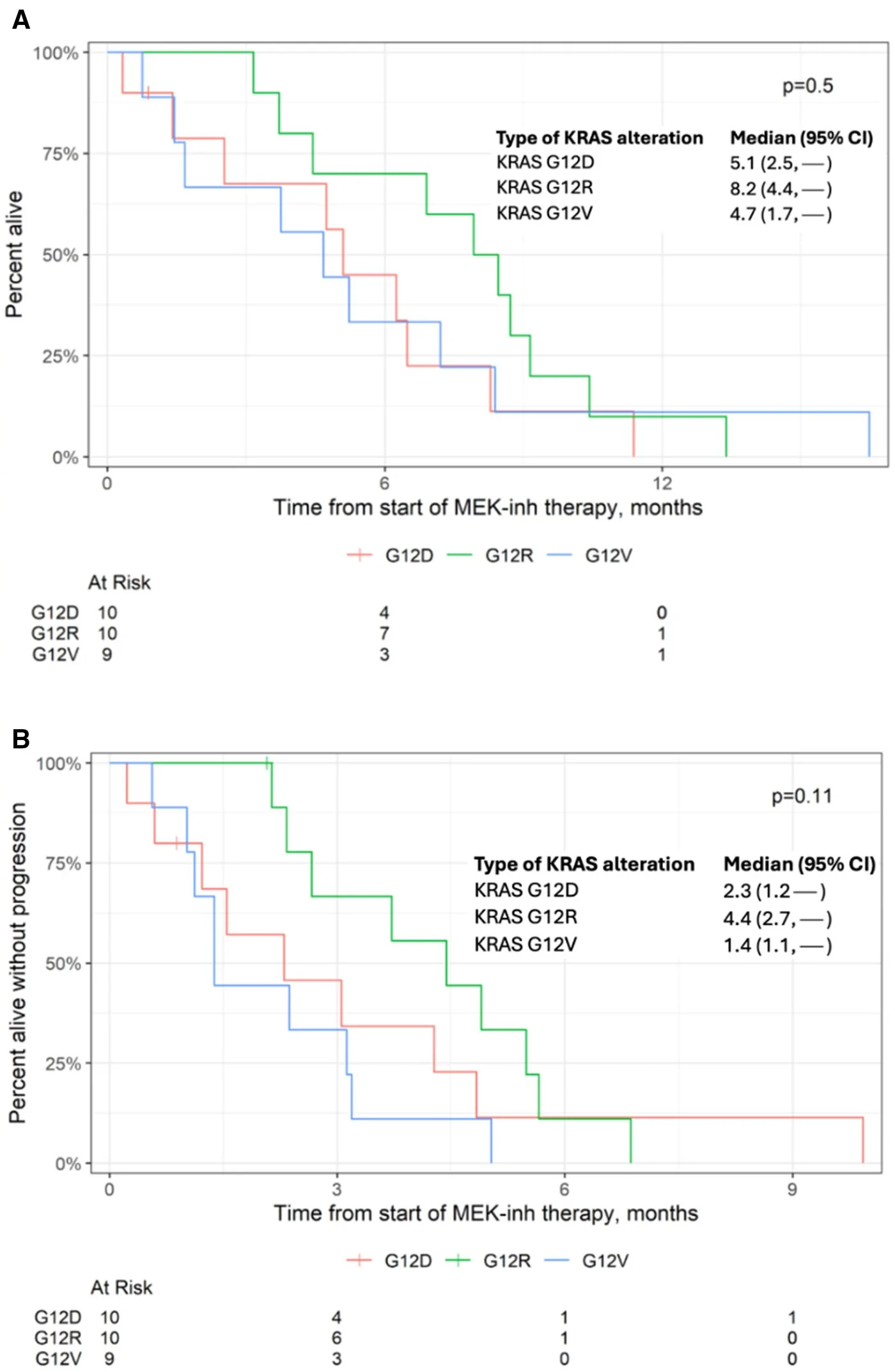
(A) Overall survival from MEK inhibitor therapy initiation. (B) Progression-free survival from time of MEKi therapy.

Median OS from MEKi therapy initiation for patients with *KRAS* G12R alterations was 8.2 months, G12D 5.1 months, and G12V 4.7 months (Figure 1A). Median PFS from MEKi therapy initiation for patients with *KRAS* G12R alteration was 4.4 months, G12D 2.3 months, and G12V 1.4 months (Figure 1B). The difference between OS and PFS for groups of patients with different *KRAS* alterations (G12R/G12D/G12V) was not statistically significant.

Fifteen patients (52%) experienced interruption of at least one drug in their treatment combination due to any level of drug–related toxicity (none grade 4 or 5). In 6 of 29 (21%) at least one drug in their treatment combination was discontinued due to any level of drug–related toxicity (none grade 4 or 5). Three patients (10%) discontinued both drugs in 2-drug combination regimens due to disease progression/clinical decline and suspected drug–related toxicities: trametinib/palbociclib due to mucositis/cytopenia (*N* = 1) and gastrointestinal discomfort (*N* = 1); trametinib/bevacizumab due to diarrhea/hypertension (*N* = 1).

## Discussion

Of the 29 patients with metastatic PDAC who were treated with MEKi combinatorial targeted therapies at our institution, 5 of 10 (50%) of patients with *KRAS* G12R were free of progression by 4 months (PFS in months among the 5 patients [line of therapy]: 6.9 [3^rd^], 5.6 [4^th^], 5.5 [1^st^], 4.9 [1^st^] and 4.4 [1^st^]), compared to 3/10 (30%) in *KRAS* G12D (PFS in months among the 3 patients [line of therapy]: 9.9 [3^rd^], 4.8 [3^rd^], 4.3 [3^rd^]), and 1/9 (11%) in *KRAS* G12V (PFS 5.03 months [2^nd^ line]).

The benchmark PFS for chemotherapy in the second line of treatment for PDAC is 3.1 months [8]. MEKi-genomically matched individualized combinatorial targeted therapies in patients with metastatic PDAC with *KRAS* alterations in our study, therefore, had modest benefit, which was most prominent in patients with *KRAS* G12R alterations. A prior study showed the clinical benefit rate (stable disease >6 months/complete response/partial response) was 80% in first line versus 8% beyond first line for similar targeted therapy in a pilot investigation in PDAC [3]. While *KRAS* G12C inhibitors are FDA approved for other cancers, the rarity of this alteration in PDAC (<1%) has limited their impact [9]. Daraxtonrasib (RMC-6236), the most clinically advanced pan-RAS(ON) inhibitor so far, showed promising PFS of 7.6 months in the second line in a phase 1 trial [10]. Whether this promising result stands in a larger population is being tested in the RASolute-302 phase 3 clinical study.

## Conclusions

While awaiting trial completion for *KRAS* inhibitors, there may be a role for MEKi combinatorial targeted therapies in patients with metastatic PDAC, specifically in those with *KRAS* G12R alteration and as earlier lines of therapy.

## Supplementary Material

oyag040_Supplementary_Data

## Data Availability

The data underlying this article will be shared on reasonable request to the corresponding author.
