# Supplementary material for: MEK inhibitor–based genomically matched combinatorial targeted therapies in metastatic pancreatic adenocarcinoma with KRAS alterations
Source: Oncologist. 2026 Feb 17;31(10):oyag040. doi: 10.1093/oncolo/oyag040 (PMC13592677; doi:10.1093/oncolo/oyag040)
Supplement: Supplementary file 1 [file oyag040_supplementary_data.docx]

| **ID#** | **Type of KRAS alteration** | **-Gender**  **-Age**  **-ECOG**  **(At MEKi initiation)** | **-Type of test (# of genes tested)**  **-Genomic Alterations** | **-Type of MEKi based therapy** | **# of Line of MEKi therapy in metastatic setting** | **Lines of treatment prior to MEKi based therapy** | **PFS from initiation of MEKi therapy (+ indicates ongoing)** | **OS from initiation of MEKi therapy** | **-Adverse event leading to treatment discontinuation of a drug (Yes/No)**  **-Drug discontinued: Toxicity leading to drug discontinuation** | **-Adverse event leading to treatment interruption of a drug (Yes/No)**  **-Drug interrupted: Toxicity leading to drug interruption** |
| --- | --- | --- | --- | --- | --- | --- | --- | --- | --- | --- |
| **KRAS G12R** | | | | | | | | | | |
| 1 | G12R | -Male  -Age 62  -ECOG 1 | -Tempus xT (648)  -**KRAS p.G12R**  TP53 p.V274D  **CDKN2A p.T79fs** | **trametinib**  **palbociclib** | 3 | Neoadjuvant  FOLFIRINOX  Metastatic  GnP  NALIRIF | 6.87 | 10.42 | -No | -Yes  -palbociclib: neutropenia |
| 2 | G12R | -Female  -Age 61  -ECOG 0 | -Tempus xT (648)  -**KRAS p.G12R**  TP53 p.W91*  **CDK2NA deletion**  MTAP del  PTPRD del | **trametinib**  **palbociclib** | 4 | Metastatic  GnP  NALIRIF  FOLFOX | 5.65 | 7.92 | -Yes  -erlotinib: nausea/vomiting | -Yes  -palbociclib: neutropenia |
| 3 | G12R | -Female  -Age 68  -ECOG 1 | -Tempus xT (648)  **KRAS p.G12R**  **TP53 p.C135fs**  **SMAD4 p.S343fs** | **trametinib**  **bevacizumab**  **erlotinib** | 1 | Neoadjuvant  FOLFIRINOX  GnP  Adjuvant  GnP | 5.49 | 13.38 | -No | -Yes  -erlotinib: diarrhea, skin rash |
| 4 | G12R | -Female  -Age 70  -ECOG 0 | -Tempus xT (648)  -**KRAS p.G12R**  **TP53 p.Q317fs** **CDKN2A p.L117P**  **SMAD4 p.G270fs** | **trametinib**  **bevacizumab**  **palbociclib**  **erlotinib** | 1 | Neoadjuvant  GnP | 4.90 | 9.14 | -No | -Yes  -erlotinib: nausea/vomiting |
| 5 | G12R | -Male  -Age 67  -ECOG 0 | -Tempus xT (648)  -**KRAS p.G12R**  TP53 R196*  **CDKN2A p.Q85*** | **cobimetinib/HCQ**  **abemaciclib** | 1 | Neoadjuvant  FOLFIRINOX  GnP | 4.44 | 4.44 | -No | -Yes  -cobimetinib: atrial flutter |
| 6 | G12R | -Female  -Age 64  -ECOG 1 | -Tempus xT (648)  -**KRAS p.G12R**  TP53 p.M237K  **CDK4 p.R24H** | **cobimetinib**  **ribociclib** | 3 | Metastatic  FOLFIRINOX  FOLFIRI | 3.72 | 3.72 | -No | -No |
| 7 | G12R | -Male  -Age 73  -ECOG 1 | -Tempus xT (648)  -**KRAS p.G12R**  **TP53 p.E298***  **ERBB2 amp** | **trametinib**  **bevacizumab**  **afatinib** | 4 | Metastatic  GnP  NALIRIF  Trastuzumab deruxtecan | 2.66 | 6.90 | -No | -No |
| 8 | G12R | -Female  -Age 68  -ECOG 1 | -FoundationOne CDx (324)  -**KRAS p.G12R**  TP53 c.933+1G>T  MLL2 c.16052+1G>C  **CDKN2A/B del**  MTAP del  GATA6 amp | **trametinib**  **palbociclib** | 5 | Metastatic  FOLFIRINOX  GnP  Capecitabine  NALIRIF | 2.33 | 3.16 | -No | -Yes  -trametinib: skin rash  -palbociclib: fatigue |
| 9 | G12R | -Male  -Age 74  -ECOG 1 | -Tempus xT (648)  -**KRAS p.G12R**  STK11 p.E57fs | **trametinib/erlotinib** | 4 | Metastatic  FOLFIRINOX  GnP  Gemcitabine/capecitabine/docetaxel | 2.14 | 8.45 | -No | -Yes  -trametinib: skin rash |
| 10 | G12R | -Female  -Age 69  -ECOG 0 | -Tempus xT (648)  -**KRAS p.G12R**  **TP53 p.D259V** | **trametinib/afatinib**  **bevacizumab** | 1 | Neoadjuvant  FOLFIRINOX | 2.07+ | 8.71 | -Yes  -Afatinib  -elevated liver enzymes | -Yes  -trametinib+ afatinib: elevated liver enzymes |
| **KRAS G12D** | | | | | | | | | | |
| 11 | G12D | -Male  -Age 51  -ECOG 1 | -FoundationOne CDX (324)  -**KRAS p.G12D**  SMAD4 p.L146*  **ARID1A p.G89fs*12** | **trametinib**  **pembrolizumab/olaparib** | 3 | Metastatic  GnP  FOLFIRI | 9.93 | 11.38 | -No | -No |
| 12 | G12D | -Female  -Age 68  -ECOG 1 | -Tempus xT (648)  -**KRAS p.G12D**  **TP53 p.S241A**  TP53 c.376-1G>A  CDKN2A p.MS3fs PPM1D p.L484* | **cobimetinib**  **bevacizumab** | 3 | Neoadjuvant  FOLFIRINOX  Metastatic  GnP/cisplatin  gemcitabine | 4.83 | 8.28 | -No | -No |
| 13 | G12D | -Male  -Age 66  -ECOG 0 | -Tempus xT (648)  -**KRAS p.G12D**  **TP53 p.590fs**  CDKN2A/B del  SMAD4 del  **MTAP del** | **trametinib**  **bevacizumab**  **pemetrexed** | 3 | Neoadjuvant  GnP  Metastatic  GnP  FOLFIRINOX | 4.27 | 6.25 | -No | -No |
| 14 | G12D | -Female  -Age 65  -ECOG 0 | -Tempus xT (648)  -**KRAS p.G12D**  **SMAD4 pY412fs** | **trametinib**  **afatinib + pertuzumab** | 2 | Metastatic  GnP + investigational drug (clinical trial) | 3.06 | 5.10 | -Yes  -afatinib: nausea/vomiting | -Yes  -afatinib: nausea/vomiting |
| 15 | G12D | -Male  -Age 63  -ECOG 1 | -Tempus xT (648)  -**KRAS p.G12D**  **TP53 p.Q317*** **CDKN2A p.E61***  FBXW7 p.R465C, BCL6 amp | **trametinib**  **bevacizumab**  **palbociclib** | 2 | Neoadjuvant  GnP  FOLFIRINOX  Adjuvant  FOLFIRINOX  Metastatic  Investigational drug (clinical trial) | 2.30 | 4.73 | -No | -No |
| 16 | G12D | -Female  -Age 80  -ECOG 1 | -Tempus xT (648)-**KRAS p.G12D**  **TP53 p.R175H**  NF2 pQ298* | **trametinib**  **bevacizumab** | 2 | Neoadjuvant  GnP  Metastatic  NALIRIF | 1.55 | 2.53 | -No | -No |
| 17 | G12D | -Female  -Age 67  -ECOG 1 | -Tempus xT (648)  -**KRAS p.G12D**  **TP53 p.G2455** | **trametinib**  **bevacizumab** | 3 | Neoadjuvant  GnP  Metastatic  GnP  NALIRIF | 1.22 | 6.48 | -Yes  -trametinib: diarrhea  -bevacizumab: hypertension | -Yes  -trametinib: diarrhea  -bevacizumab: hypertension |
| 18 | G12D | -Female  -Age 75  -ECOG 2 | -Tempus xT (648)- **KRAS p.G12D**  TP53 p.V157F  **SMAD4 p.K385*** | **trametinib**  **afatinib** | 1 |  | 0.89 | 0.89 | -No | -No |
| 19 | G12D | -Female  -Age 45  -ECOG 1 | -Tempus xT (648)**-KRAS p.G12D**  TP53 p.E298*  **CDKN2A p.H83D**  ARID1A p.Q512fs CARM1 amp  CCNE1 amp | **trametinib**  **palbociclib** | 2 | Metastatic  NALIRIF | 0.59 | 1.41 | -Yes  -trametinib/palbociclib: GI discomfort | -Yes  -trametinib/palbociclib: GI discomfort |
| 20 | G12D | -Female  -Age 68  -ECOG 2 | -Tempus xT (648)  -**KRAS p.G12D**  TP53 p.V173fs **CDKN2A p.M53V** | **trametinib**  **palbociclib** | 3 | Metastatic  GnP + investigational drug (clinical trial)  FOLFIRINOX | 0.23 | 0.33 | -No | -No |
| **KRAS G12V** | | | | | | | | | | |
| 21 | G12V | -Female  -Age 67  -ECOG 1 | -STRATA (429)  -**KRAS p.G12V**  **CKDN2A p.M9Ifs*6** | **trametinib**  **palbociclib** | 2 | Metastatic  FOLFIRINOX | 5.03 | 16.47 | -No | -Yes  -palbociclib: neutropenia |
| 22 | G12V | -Female  -Age 66  -ECOG 1 | -Tempus xT (648)  -**KRAS p.G12V**  TP53 p.C124R  **CDKN2A p.H83N**  **SMAD4 p.5178**  ELF3 p.T129fs  EGFR p.L730R | **cobimetinib**  **palbociclib**  **erlotinib** | 3 | Neoadjuvant  FOLFIRINOX  Metastatic  GnP  gemcitabine/erlotinib | 3.19 | 4.67 | -No | -Yes  -palbociclib: neutropenia  -trametinib: lower extremity edema |
| 23 | G12V | -Female  -Age 75  -ECOG 1 | -FoundationOne CDX (324)  -**KRAS p.G12V**  TP53 p.D281H  **CDKN2A/B del**  ARID1A p.E1542*  ERBB4 P800S  NSD3 amp | **trametinib**  **palbociclib** | 4 | Metastatic  FOLFIRINOX  GnP  gemcitabine/carboplatin | 3.12 | 8.38 | -Yes  -trametinib: mucositis  -palbociclib: cytopenia | -Yes  -trametinib: mucositis  -palbociclib: cytopenia |
| 24 | G12V | -Male  -Age 59  -ECOG 1 | -Tempus xT (648)-**KRAS p.G12V**  TP53 p.S116fs **CDKN2A p.G45fs**  MSH3 p.S762fs | **trametinib**  **bevacizumab**  **abemaciclib** | 1 |  | 2.37 | 7.20 | -No | -No |
| 25 | G12V | -Female  -Age 78  -ECOG 1 | -Tempus xT (648)-**KRAS p.G12V**  TP53 p.L194R  **SMAD4 p.Q28*** | **trametinib**  **afatinib** | 3 | Neoadjuvant  FOLFIRINOX  Metastatic  GnP  NALIRIF | 1.38 | 3.75 | -No | -Yes  -trametinib/afatinib: mucositis |
| 26 | G12V | -Male  -Age 77  -ECOG 0 | -FoundationOne CDX (324)  -**KRAS p.G12V**  **TP53 p.V272M**  CDKN2A rearrangement intron 1  NF1 p.W1662*  CNNE1 amp | **trametinib**  **bevacizumab**  pembrolizumab | 5 | Metastatic  GnP  NLIRIF  FOLFOX  Capecitabine | 1.38 | 1.68 | -No | -No |
| 27 | G12V | -Male  -Age 74  -ECOG 1 | -Tempus xT (648)  -**KRAS p.G12V**  **BRCA p.E23fs**  CDKN2A p.R58* | **trametinib**  **pembrolizumab/Olaparib** | 3 | Neoadjuvant  FOLFIRINOX  Metastatic  gemcitabine/cisplatin  olaparib | 1.12 | 5.23 | -No | -No |
| 28 | G12V | -Male  -Age 52  -ECOG 1 | -Tempus xT (648)  **-KRAS p.G12V**  **TP53 p.S241fs**  **CDKN2A p.H83R**  FGF4 amp  KMT2C amp  SMAD4 amp | **trametinib**  **bevacizumab**  **palbociclib** | 4 | Metastatic  FOLFIRINOX  GnP  GnP/cisplatin | 1.02 | 1.35 | -No | -No |
| 29 | G12V | -Male  -Age 48  -ECOG 1 | -Tempus xT (648)  -**KRAS p.G12V**  TP53 p.Y205*  **CDKN2A/B del**  BTG1 del  MTAP del | **trametinib**  **palbociclib** | 3 | Metastatic  FOLFIRINOX  GnP | 0.56 | 0.76 | -No | -No |

Abbreviations: Amp: amplification; Del: deletion; FOLFIRI: folinic acid + fluorouracil + irinotecan; FOLFIRINOX: folinic acid + fluorouracil + irinotecan + oxaliplatin; FOLFOX: folinic acie + fluorouracil + oxaliplatin; HCQ: hydroxychloroquine; ID#: unique identifier; MEKi: MEK inhibitor; NALIRIF: liposomal irinotecan + fluorouracil; GI: gastrointestinal; GnP: gemcitabine + nab-paclitaxel; GnP/cisplatin: gemcitabine + nab-paclitaxel + cisplatin; PFS: progression-free survival; OS: Overall survival.
